# Control of the Spin Angular Momentum and Orbital Angular Momentum of a Reflected Wave by Multifunctional Graphene Metasurfaces

**DOI:** 10.3390/ma11071054

**Published:** 2018-06-21

**Authors:** Chen Zhang, Li Deng, Jianfeng Zhu, Weijun Hong, Ling Wang, Wenjie Yang, Shufang Li

**Affiliations:** Beijing Key Laboratory of Network System Architecture and Convergence, Beijing Laboratory of Advanced Information Network, Beijing University of Posts and Telecommunications, 10 Xitucheng Road, Beijing 100876, China; zhangchenzc@bupt.edu.cn (C.Z.); zhujianfeng@bupt.edu.cn (J.Z.); hongwj@bupt.edu.cn (W.H.); lingwang@bupt.edu.cn (L.W.); yangwenjie07@outlook.com (W.Y.)

**Keywords:** graphene, metasurface, spin angular momentum, orbital angular momentum, terahertz

## Abstract

Three kinds of multifunctional graphene metasurfaces based on Pancharatnam–Berry (PB) phase cells are proposed and numerically demonstrated to control a reflected wave’s spin angular momentum (SAM) and orbital angular momentum (OAM) in the terahertz (THz) regime. Each proposed metasurface structure is composed of an array of graphene strips with different deviation angles and a back-grounded quartz substrate. In order to further help readers have a deeper insight into the graphene-based metasurfaces, a detailed design strategy is also provided. With the aid of the designed graphene elements, the proposed metasurfaces can achieve the full 360° range of phase coverage and provide manipulation of SAM and OAM of a circularly polarized (CP) wave at will. More importantly, simultaneous control of these two momentums can also be realized, and in order to demonstrate this function, a THz spin-controlled OAM beam generator with diverse topological charges is created, which can provide one more degree of freedom to improve the channel capability without increasing the bandwidth compared to a linearly polarized (LP) OAM beam. Numerical results verify the proposed graphene metasurfaces, which pave the way for generating spin OAM vortex waves for THz communication systems.

## 1. Introduction

In recent years, controlling spin angular momentum (SAM) and orbital angular momentum (OAM) of the electromagnetic waves at will has attracted considerable attention from the scientific community due to the unprecedented potential of polarization multiplexing [1,2] for communication systems. These two momentums are characteristic properties of a spreading wave and are associated with the polarization and phase of the electromagnetic field. As one of the most effective methods, the metasurface [3,4,5,6,7,8,9,10,11,12,13,14,15,16,17,18,19,20,21,22,23,24,25,26,27,28,29], a new kind of two-dimensional equivalence of metamaterials, has been proposed to produce abrupt phase shifts, realizing arbitrary control of polarization and phase. Due to the flexibility in controlling the wavefront, the metasurface has wide applications, including anomalous reflection/refraction [3,4,5,6], beam split [7,8,9], reflect-/transmit-array [10,11,12], vortex beam [13,14,15,16,17,18,19,20,21], photonic Spin Hall effect [22,23,24,25,26], polarization converter [27,28,29], absorber [30,31], radar cross-section reduction [32,33], imaging [34,35] and illusion [36]. However, most metasurfaces are structured by metallic cells, which possess high losses due to the skin effect in the THz band, and few reach a 360° phase adjustment, affecting its accuracy. Therefore, the fact that arbitrarily manipulating SAM and OAM of an electromagnetic wave by the metasurface causes reasonable losses for THz applications is a challenging problem.

Fortunately, graphene, a honeycomb-like structure of monolayer carbon atoms, has been found and viewed as a promising material for THz components and applications. Due to the unique monolayer structure, graphene has a weak interaction with incident waves and supports the spreading of surface plasmon polaritons (SPPs), which leads to a smaller surface loss compared with metallic metasurfaces in the THz regime. Furthermore, different from metallic cells depending on geometric variations, the electromagnetic responses of graphene elements can be tuned by chemical doping or electrical gating [37]. In virtue of the distinctive properties and outstanding tunability, graphene is increasingly applied to metasurfaces, and diverse functions are also being designed and evidenced. Eduardo et al. proposed a fixed-beam reflect-array antenna [38] based on graphene patches in the THz frequency band, which achieved good performance and grating lobe suppression, but possessed a smaller size than traditional metallic reflect-arrays under the same conditions. Then, Liu et al. designed a graphene metasurface with long-/short-strip resonators [39] to implement dynamic phase modulation and beam steering by applying different voltages; moreover, this configuration has a high reflection efficiency and a broad operation band. On the basis of the graphene elements, Shi et al. developed a wideband tunable reflect-array composed of complex graphene metamaterials to generate an OAM vortex wave with various modes [40]. In order to meet the needs of applications with various polarizations, Chen et al. proposed a wideband tunable cross polarization converter using a hollow-carved H array [41], exhibiting a circularly polarized ratio of over 95% circularly polarizedand a bandwidth of approximately 3 THz. Chen et al. proposed a 99.5% efficiency graphene absorber [42] with the capability of dual-frequency and 2.7 THz broadband absorption. Some newly emergent functionalities of graphene plasmonic amplitude/phase modulators has enriched the field of metasurfaces, such as in analog computing and beam manipulation. Sajjad et al. proposed a transmit-array of graphene-based metalines to realize analog computing [43], possessing a smaller structure and higher precision. This result may lead to remarkable improvements in light-based plasmonic signal processors at nanoscale instead of bulky conventional dielectric components. In addition, their team also proposed gate-tunable graphene-based transmit-array [44] to realize focusing and splitting, which are promising for nano-photonic and optoelectronic applications. The aforementioned graphene metasurfaces have exhibited diverse functions by controlling phases, amplitudes and polarizations of waves; however, few designs can achieve full 360° phase adjustment only by changing the geometric parameters or chemical potentials of the graphene in the THz regime. As a result, the deviation of phase control will undermine the performance and accuracy for controlling SAM and OAM of the electromagnetic waves in the THz regime.

**Figure 1:**
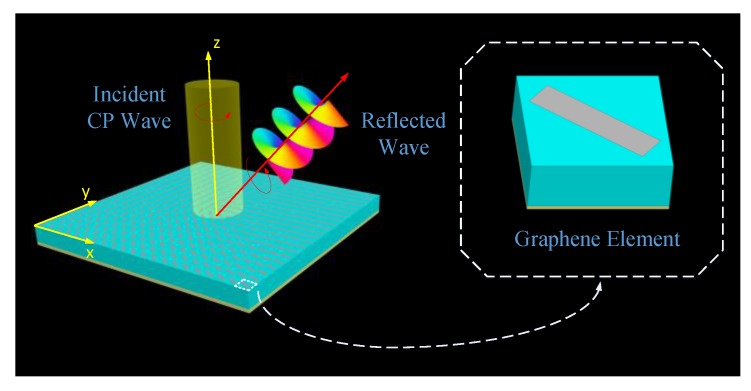
The scheme of the proposed multifunctional graphene metasurface. When a circularly polarized (CP) wave is normally incident, the metasurface can control the spin angular momentum (SAM) or/and orbital angular momentum (OAM) of the reflected wave. The inset shows the structure of a graphene element.

In this paper, we propose three kinds of multifunctional graphene metasurfaces based on the PB phase to arbitrarily control the SAM and OAM of a THz reflected wave, as shown in Figure 1. Each proposed design consists of an array of graphene elements with specific deviation angles that conform to the PB phase. In order to further help give readers a deeper insight into the graphene-based metasurfaces, a detailed design strategy is also provided. With the help of the designed cells, the proposed metasurfaces can manipulate the phase and polarization of reflected beams at will. More importantly, simultaneously controlling the polarizations and wavefronts of the reflected waves can also be realized simply by rotating the orientations of the graphene elements; to demonstrate this, a THz spin-controlled OAM beam generator with arbitrary topological charges is created. Compared with a linearly polarized OAM beam, our proposed circularly polarized spin-controlled beams provide an extra degree of freedom, improving the channel capability without increasing the bandwidth for a THz wireless communication system.

This paper is organized as follows: in Section 2, the design, theory, and simulation of the unit structure are presented. In this part, the characteristics of graphene are firstly introduced in Section 2.1, and then the design parameters and simulation response of the graphene-based unit cell are provided in Section 2.2. To guide researchers in designing these kinds of metasurface, a detailed design strategy is supplied in Section 3. With the proposed unit cell and design strategy, three kinds of graphene-based metasurfaces, with capabilities including the control of SAM, OAM and both, are discussed in Section 4.1, Section 4.2 and Section 4.3, respectively. Finally, the conclusion and contribution of the paper are provided in Section 5. 

## 2. Unit Structure Design, Theory, and Simulation

### 2.1. The Characteristics of Graphene

**Figure 2:**
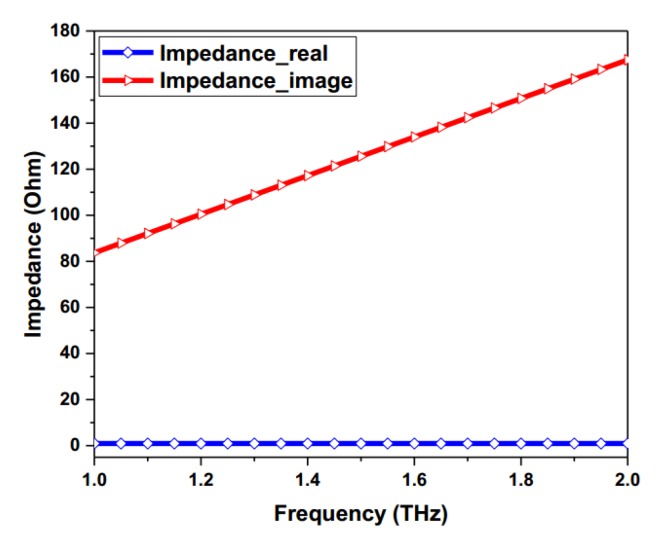
Impedance curve of the graphene with the specified parameters. The real part and imaginary part of the graphene’s impedance vary in the range of the 1 ~ 2 THz frequency band.

The conductivity of graphene is characterized by the sum of intra-band conductivity $\sigma_{\mathrm{intra}}$ and inter-band conductivity $\sigma_{\mathrm{inter}}$. However, when the graphene is under the conditions of low THz operation frequency and room temperature, the inter-band conductivity can be neglected, and the conductivity of the graphene is mainly determined by the intra-band conductivity. Considering its one-atom thickness, the surface conductivity σ of graphene in the intra-band can be calculated by the Drude model [45]:

(1) $$\sigma_{\mathrm{intra}}(\omega ,\mu_{c},\Gamma ,T)=-j\frac{e^{2}k_{B}T}{\pi \hbar^{2}(\omega -j\tau^{-1})}(\frac{\mu_{c}}{k_{B}T}+2\ln(e^{-\frac{\mu_{c}}{k_{B}T}}+1)),$$

where e is the elementary charge, $k_{B}$ is the Boltzmann’s constant, ℏ is the reduced Plank’s constant, T is the room temperature, τ is the relaxation time, ω is the radian frequency, and $\mu_{c}$ is chemical potential. In our design, the room temperature T is 300 K, the chemical potential $\mu_{c}$ = 0.64 eV [46] and the carrier mobility (μ) of 230,000 cm^2^/(V·s) are assumed [47]. The surface impedance of the graphene can be calculated as $Z\approx 1/\sigma_{\mathrm{intra}}$. As depicted in Figure 2, the real part of its impedance changes very little, while the imaginary part exhibits a linear property as the frequency increases.

### 2.2. The Design and Simulation Response of the Graphene-Based Unit Cell

Phase and polarization are important features for controlling electromagnetic waves, and they directly affect the SAM and OAM of radiated beams, respectively. Based on the PB phase principle, metasurfaces can allow variations in polarizations and phases only by rotating the angles of the elements. The PB phase [48] cells can be designed by using subwavelength scatters with identical geometric parameters but spatially varying orientations. More importantly, a PB phase cell can realize a phase 2φ only by rotating an orientation angle of φ, which greatly reduces the complexity of the scattering units. Moreover, this method can cover a 360° adjustable phase range, and it enables the design to possess a wide operation band. Therefore, the PB phase, as a high-efficiency approach associated with polarization change, has been used to design graphene elements for achieving the desired phases.

**Figure 3:**
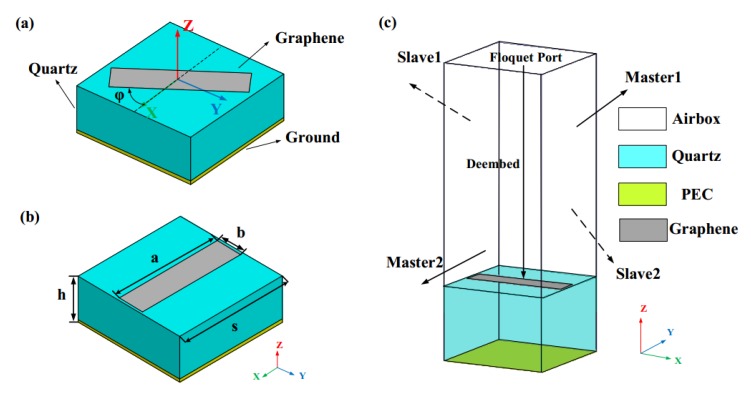
Illustration of the proposed graphene meta-atom: (**a**) Structure of the graphene element when it is rotated at an orientation angle φ with respect to the *x*-axis; (**b**) the parameters of the graphene element; (**c**) simulation setup.

Different from the traditional meta-atoms of metasurfaces, which introduce abrupt phase jumps by changing the geometric parameters of cells, the proposed graphene element can easily achieve the desired phase of 2φ by rotating an orientation angle of φ thanks to the PB phase principle, as depicted in Figure 3a. This method not only reduces the complexity of the design greatly, but also provides a full 360° range of phase coverage in the THz frequency band, which is of great importance to manipulating phase fronts accurately, especially when producing OAM beams or performing a focusing function. As the basic elements of the proposed reflective-type metasurface, each element consists of a rectangular strip with a length of 13.39 μm (*a*) and a width of 3.2 μm (*b*), deposited on the square element with sides of 14 μm (*s*). The thickness of the back-metalized quartz substrate is 26 μm (*h*), the relative permittivity is 3.75 ($\varepsilon_{r}$) [38] and the loss tangent is 0.0184 (tanδ), as shown in Figure 2b. It is worth noting that quartz is birefringent at THz frequencies; however, here the amorphous quartz glass (SiO_2_) [49] is used to ensure the correctness of the design. In the simulation, two groups of periodic boundary conditions are assigned to the four walls along the x and y directions to simulate an infinite metasurface. In addition, as depicted in Figure 2c, a “Floquet” port is used to excite the element and the “De-embed” condition is selected in the software HFSS. Generally, the infinite metasurface design is simulated by using the Floquet port method, which consists of only a single unit cell and applies the appropriate boundary conditions for assuming a planar, infinite array. Because the reference plane of reflection phase is the metasurface, here, a “De-embed” condition that changes the reference plane where the port is located should be set. 

**Figure 4:**
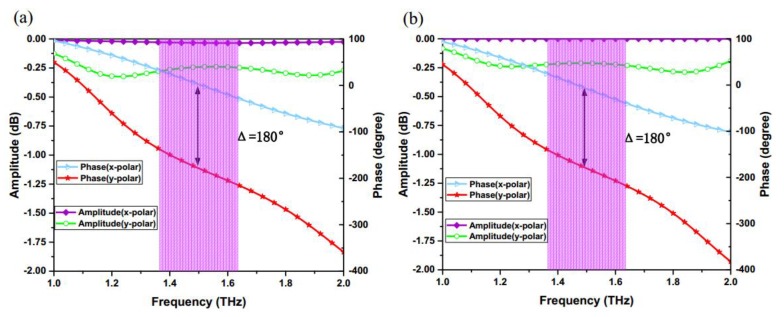
Reflection phases and reflection amplitudes of the proposed graphene element by using HFSS and CST: (**a**) the proposed graphene element achieves a 180° ± 10° reflective phase difference between x and y linearly polarized waves in the range of 1.36 ~ 1.62 THz, while reflective magnitudes are larger than −0.35 dB by using HFSS; (**b**) the proposed graphene element achieves a 180° ± 10° reflective phase difference between x and y linearly polarized waves, also in the range of 1.36 ~ 1.62 THz, but reflective magnitudes are large than −0.3 dB by using CST, and the reflection amplitude is a little larger than the amplitudes in HFSS.

On the basis of the graphene and PB phase method, in order to realize the manipulation of the incident CP wave, each element designed with graphene should possess an approximately 180° phase difference between x and y linearly polarized waves. Meanwhile, considering the influence of graphene surface losses, reflection coefficients for two polarized waves should be controlled to within an acceptable range. As depicted in Figure 4a, the proposed graphene elements achieve a 180° ± 10° reflective phase difference between x and y linearly polarized waves in the range of 1.36 ~ 1.62 THz, while reflective magnitudes are larger than −0.35 dB. Here, the optimized parameters are selected in our structure, as described in Figure 3b, which ensures the proposed unit produces a good phase and amplitude responses for the incident CP wave. The reflective phase of this structure can cover 360° range, which provides a possibility for accurately performing a variety of different functions, such as the generation of vortex beams.

In order to verify the results of the unit cell in HFSS, another modeling tool CST has also been used to simulate the same unit cell, as shown in Figure 4b. By comparing the two results, the trends of cures on phase and amplitude are similar but a little difference under the condition of same parameters. The reasons caused the difference may come from two factors. On the one hand, the difference of algorithms in two simulation tools may cause the difference of the results. The HFSS software uses the Finite Element Method (FEM) algorithm, while the CST software adopts the Finite Integration (FIT) algorithm. The FIT algorithm is fast, but less accurate than FEM for electric small-size objects [50]. On the other hand, the different representations of graphene in two modeling tools may also influence the results. In HFSS, an impedance boundary condition with variables is used to represent the graphene. However, in CST (after the 2016 version), the graphene material is defined by a graphene macro instead of the former. Therefore, based on two factors and the corresponding simulation results, the simulation results by HFSS can be verified by another computational tool and have a higher precision.

**Figure 5:**
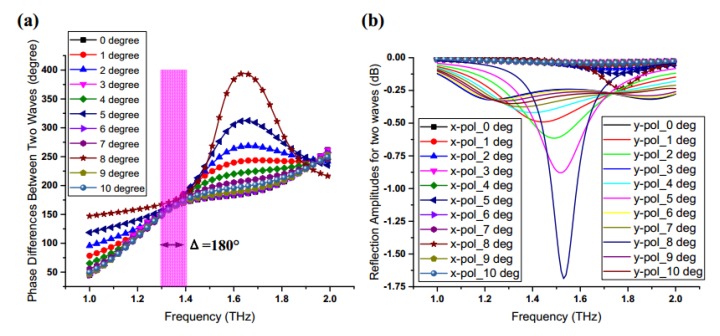
Reflection phase differences and the reflection amplitudes between x- and y-polarized waves under the illumination with different incident angles: (**a**) the phase difference curve varies with changing the incident angle and the operation bandwidth reduces to the range of 1.3 ~ 1.4 THz; (**b**) reflection amplitudes of x- and y-polarized waves change with variation in the incident angle in the range 0 ~ 10 degrees.

In this proposal, although normal illumination is used as the main way, an oblique incidence is also allowable. However, due to the strict condition of co-polarization conversion, which requires that the phase difference between x-polarized wave and y-polarized wave approach 180° ± 10°, the bandwidth will reduce greatly as the oblique incident angle increase. Through the simulation validation, we found that the range of oblique incidence was only within 10° for the graphene-based unit cell, while the operation bandwidth that approaches 180° ± 10° phase difference decreases to 0.1 THz (1.3 ~ 1.4), as shown in Figure 5a, and its variation of reflection amplitude when the incident angle changes from 0° to 10° is as depicted in Figure 5b. 

In order to observe the variation of reflection amplitude and phase when the incident wave changes from 0° to 10°, the related simulation results are provided as follows. The phase differences and reflection amplitudes between two line-polarized waves under the different incident angles are depicted in Figure 5a,b, respectively. It can be seen that the range of oblique incidence is only within 10° for the graphene-based unit cell, while the operation bandwidth that approaches 180° ± 10° phase difference decreases to 0.1 THz (1.3 ~ 1.4 Thz). In addition, the difference between the two kinds of reflection amplitudes will increase with variation of the incident angles. 

Not only can the way of normal illumination provide a wider bandwidth (1.36 ~ 1.62 THz), but it also ensures the co-polarization conversion of the reflected wave. On the other hand, some references suggest that an offset feeding configuration should be avoided, because an amplitude null is in the center of the vortex beam of OAM. This is the smallest blocking effect on the reflective OAM vortex wave using normal incidence [51,52]. Considering two factors, we only use normal illumination as the main way of excitation. We will also exploit new methods to further improve our design in future.

## 3. Design Strategy

In order to further provide readers a deeper insight into graphene-based metasurfaces and provide more people with guidelines for the design of our proposals, a design strategy will be given in more detail. The design steps are as follows.

(1) Firstly, the operation frequency band and the characteristics of the graphene should be considered. The conductivity of graphene includes two parts, i.e., the intra-band part and the inter-band part, and the influence of these on graphene is different in the low-frequency band and the high-frequency band. In our design, a low-frequency band (1.36–1.62 THz) is chosen; therefore, the conductivity of graphene is only determined by the intra-band part. By using Equation (1), a complex conductivity $\sigma_{\mathrm{intra}}$ can be obtained.
(2) Secondly, a unit cell composed of the graphene strip and a quartz substrate backed with a metal ground should be modeled in the HFSS software. In the simulation environment, a strip with an impedance boundary condition is adopted to represent the graphene strip. Due to the fact that the graphene’s impedance can be expressed by its conductivity, i.e., $Z=1/\sigma_{\mathrm{intra}}$, the graphene can be accurately represented. Two pairs of periodic boundary conditions (master/slave) are assigned to the four walls of the model to simulate an infinite metasurface. In addition, a “Floquet” port is used to provide the excitation and a “De-embed” condition is also used to ensure the accurate reflection phase can be achieved. The configuration of the graphene-based unit cell is accomplished.
(3) After that, parameter optimization should be executed during the simulation. The size parameters of the unit cell will not be adjusted until the phase difference between the x-polarized wave and the y-polarized wave is approximate 180° ± 10°, which is a necessary condition for realizing co-polarized conversion of reflected waves. Meanwhile, the reflection amplitude is also considered during the optimization so as to ensure the reflection efficiency. Once the aforementioned conditions have been created, the unit cell can act as the basic element for the graphene metasurface.
(4) Once the unit cell has been designed, the phase distribution of the whole metasurface should be taken into account. The function of the metasurface is mainly determined by the phase distribution of the unit cells, and the values of phase distribution can be calculated by Matlab.
(5) According to the phase distribution of the metasurface, the required rotation angles of graphene strips in corresponding positions can be obtained with the aid of the PB phase method.
(6) Finally, the target is to build up the objective metasurface using graphene strips with specific rotation angles. In the design process, the utilization of scripts and the application programming interface (API) in HFSS can greatly accelerate the speed of establishing models.

## 4. Results and Discussion

The structure topology and electromagnetic properties of the proposed graphene element have been comprehensively discussed; in addition, a design strategy was also provided to guide researchers in effectively designing a graphene-based metasurface. Using this kind of reflective-type cell, multifunctional graphene metasurfaces with the capability of controlling the SAM and OAM of reflected beams at will can be built up. To further elaborate the function and physical mechanism of the proposed graphene metasurfaces, in the following parts, three functions, including arbitrary control of SAM and OAM for a reflective wave and generation of a spin-controlled OAM helical wave, will be discussed in detail.

### 4.1. Control of the SAM of a Reflected Wave

The SAM is a momentum that characterizes the spin state of a spreading wave and is directly associated with the polarization of the wave. Circularly polarized modes carry a spin angular momentum of ±ℏ per photon [53]. In order to effectively control the SAM, an accurate manipulation of the compensation phase for a reflective interface is indispensable. Therefore, a graphene metasurface built up with PB phase elements is designed, which functions as an artificial interface by introducing discontinuous phase jumps to change the phase distribution. As a result of the optimization, each graphene PB phase cell with specific parameters described in part two has an approximately 180° phase difference for two orthogonal polarized waves (x- and y-polarized waves); meanwhile, the reflection amplitudes of the two polarizations should be close. As a result of providing a 180° phase compensation, the polarization of the reflected wave is consistent with the polarization of the incident wave. Therefore, the polarization of the reflected wave can be controlled by selecting different CP waves as the excitations. 

After the basic elements are designed, the phase distribution of the whole graphene metasurface should be determined by its function. Here, we enable the metasurface to have the capability of controlling the SAM of a reflected wave in any expected directions. According to generalized Snell’s law, abrupt phase gradients are introduced on the metasurface to realize the arbitrary beam deflection. The surface reflection phase distribution [8] can be calculated by

(2) $$\Phi (x,y)=\Phi_{0}(x,y)-k_{0}\sin\theta (x\cos\varphi +y\sin\varphi ),$$

where $k_{0}$ is the wave number in free space, Φ(x,y) and $\Phi_{0}(x,y)$ are the required phase distribution for deflection and the initial phase, θ and φ are the elevation angle and azimuth angle, respectively.

**Figure 6:**
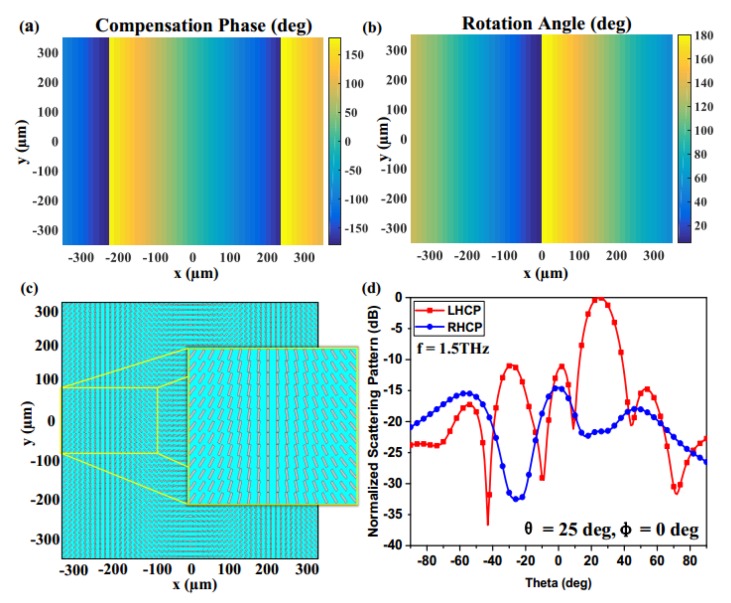
Simulation results for controlling the SAM of the reflected wave at (θ, φ ) = (25°, 0°): (**a**) Compensation phase pattern of the graphene metasurface; (**b**) rotation angle pattern of the graphene metasurface; (**c**) the element pattern of the metasurface; (**d**) 2D normalized scattering pattern of the graphene metasurface.

**Figure 7:**
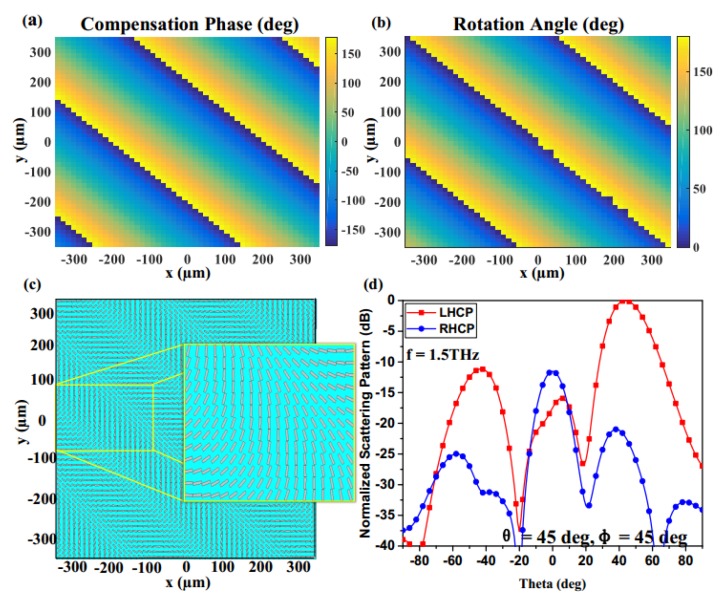
Simulation results for controlling the SAM of the reflected wave at (θ, φ ) = (45°, 45°): (**a**) Compensation phase pattern of the graphene metasurface; (**b**) rotation angle pattern of the graphene metasurface; (**c**) the element pattern of the metasurface; (**d**) 2D normalized scattering pattern of the graphene metasurface.

According to Equation (2), the compensation phase distribution of each cell can be obtained. For a phase 2φ, an element need only rotate an orientation angle for the graphene strip. To demonstrate the control of SAM for the reflected wave, two graphene metasurfaces with 51 × 51 elements were created to realize the SAM beam steering in different directions ((θ, φ) = (25°, 0°) and (θ, φ) = (45°, 45°)) under the condition of a LHCP (left-handed circularly polarized) wave normally illumination, as shown in Figure 6c and Figure 7c. In the case of (θ, φ) = (25°, 0°), the compensation phase pattern and the rotation angle pattern are depicted in Figure 6a,b, respectively, which demonstrates that the full 360° phase can be achieved only by the elements’ rotation within a 180° range. With the observation of the 2D normalized scattering pattern in Figure 6d, it is obvious that the polarization of the reflected wave is LHCP and the deflection angle of the beam points to (θ, φ) = (25°, 0°). Similarly, the simulation results in Figure 7 also verify the SAM beam steering in the (θ, φ) = (45°, 45°). The proposed graphene metasurfaces have the advantages of good performance in the THz regime, and this design method also reduces the complexity of the design greatly. In addition, the SAM of the reflected wave can be manipulated arbitrarily by varying differently polarized excitations, which is promising for the Spin Hall effect and the polarization converter. 

It is noted that the metasurface is different from the reflect-array antenna because the metasurface has no port. Therefore, we only consider its aperture efficiency, and the aperture efficiency of the metasurface approaches 44% by calculation. Because the aperture area of graphene metasurface is small and comparable to the wavelength, other kinds of metasurface aperture efficiencies are basically over 40%.

The metasurface depends on the abrupt phase shifts provided by unit cells to manipulate the incident waves and reflected waves. Given that the discrete amplitude distribution is analogous to a non-blazed grating, it is not surprising that the angular spectrum may include one or more diffracted orders [54]. The resulting discrete weight distribution produces a single beam, but with an increase in the overall side lobe levels. Depending on the application, the sidelobe levels associated with this straightforward design may be acceptable, but can certainly be decreased using other optimization methods [55]. Therefore, some optimization methods can be taken to further depress the side lobes and improve the efficiency.

### 4.2. Control of the OAM of a Reflected Wave

OAM, according to the knowledge of optics, is in relation to a helical wavefront of a spreading wave that dependents on the phase factor $e^{-jl\varphi }$, l, called topological charge, and φ, which is the azimuthal angle. Such vortex beams carry an average of lℏ orbital angular momentum per photon. In general, Laguerre—Gaussian (LG) beam [56] is a typical case. OAM is an important technology, which can add an extra dimension of polarization to a traditional communication system. This technology has wide applications, including microparticle control [57] and communications [58]. For the generation of a vortex wave carrying OAM, a desired helical phase distribution with corresponding topological charges should be patterned on the entire metasurface. The required compensation phase distribution [59] of the metasurface can be obtained by

(3) $$\left\{ \begin{array}{l} \Phi (x,y)=l\varphi \\ \varphi =\arctan(\frac{y}{x}) \end{array}\right.,$$

where Φ(x,y) is the phase distribution for each element, and φ is the azimuth angle of a position. The l is the topological charge, and (x, y) is the coordinate position of a point.

**Figure 8:**
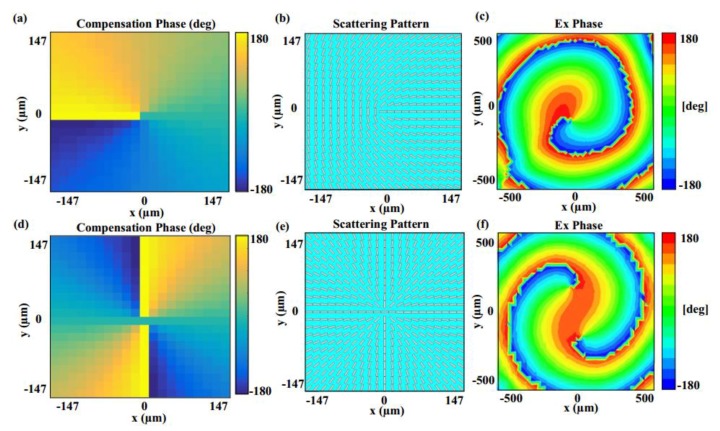
Simulation results with different topological charges: (**a**–**c**) The required compensation phase distribution, scattering pattern of the proposed metasurface and Ex phase distribution for the case L = 1; (**d**–**f**) the required compensation phase distribution, scattering pattern of the proposed metasurface and Ex phase distribution for the case L = 2.

To verify the output beam carrying OAM with desired topological charges, a plane RHCP (right-handed circularly polarized) wave as an incident wave is illuminated normally on the proposed metasurface with 21 × 21 elements. For the case of L = 1 in Figure 8a,b, the phase distribution of the metasurface experiences the phase variations from −π to π. As the L increases, the number of cycles also increases, as depicted in Figure 8d,e. According to the simulation results on the observation plane, as depicted in Figure 8c,f, the Ex phase of L = 1 will experience 2π phase variations with one helical arm from counterclockwise direction, while the phase of L = 2 experiences 4π phase variations with two helical arms, which both demonstrate the metasurfaces effectively generating OAM beams with diverse topological charges. In theory, the OAM technology can provide countless kinds of polarizations by adjusting the topological charges, and different polarizations are orthogonal with each other. Therefore, transmission of information by using this kind of metasurface can further improve the capability of a channel without increasing bandwidth. Furthermore, the proposed designs also can be used as diversity antennas for the THz massive multiple-input multiple-output (MIMO) systems to improve the isolation between different polarizations. 

### 4.3. A THz Spin-Controlled OAM Beam Generator with Arbibitrary Topological Charges

Due to the capability of controlling the SAM and OAM of a reflected wave arbitrarily by using the proposed multifunctional graphene metasurfaces, manipulating the two momentums simultaneously becomes possible. Therefore, a THz spin-controlled OAM beam generator with varying topological charges can also be created. To perform this function, the required phase distribution can be obtained by adding the two kinds of phase distributions described above, and can be expressed by

(4) $$\Phi (x,y)=l\times \arctan(\frac{y}{x})-k_{0}\sin\theta (\mathrm{xcos}\varphi +\mathrm{ysin}\varphi ),$$

where Φ(x,y) is the phase distribution of the metasurface, and $k_{0}$ is the wave number in free space. The θ and φ are the elevation angle and the azimuth angle, respectively. The l is the topological charge, and (x, y) is the coordinate position.

**Figure 9:**
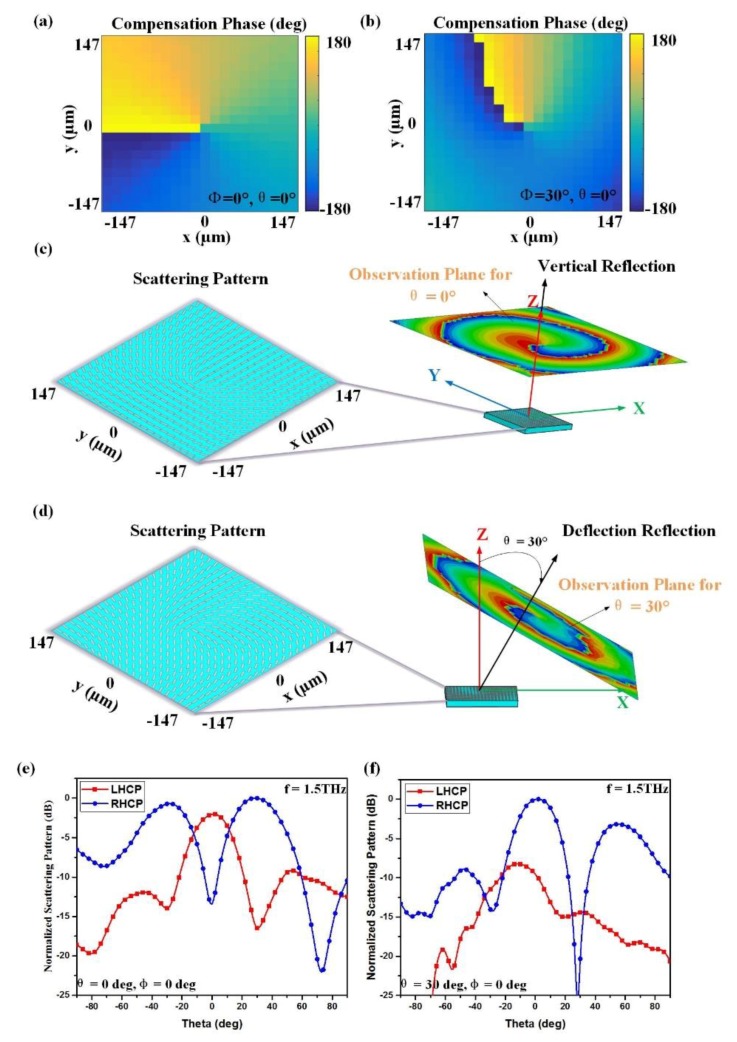
Simulation results for generating a spin-controlled OAM beam with L = 1 in arbitrary directions: (**a**,**c**,**e**) The required compensation phase distribution, scattering pattern of the metasurface and normalized scattering pattern in (0°, 0°) direction; (**b**,**d**,**f**) the required compensation phase distribution, scattering pattern of the metasurface and normalized scattering pattern in (30°, 0°) direction.

Based on the designed graphene elements, two graphene metasurfaces composed of 21 × 21 cells are proposed and analyzed, which can both convert a circularly polarized incident wave into a spin-controlled vortex wave that possesses SAM and OAM simultaneously in desired directions. In order to demonstrate the functions of the proposed metasurfaces, the RHCP plane waves, with excitations 15 wavelengths away from the metasurface, are illuminated normally on the surfaces. In Figure 9a,b, the required compensation phase distributions in (θ,φ) = (0°, 0°) and (θ,φ) = (30°, 0°) can be observed, respectively, while their phases both experience phase variations from −π to π. For the case of vertical reflection in (θ,φ) = (0°, 0°), its scattering pattern and phase distribution with L = 1 are shown in Figure 9c, which demonstrates the generation of an OAM beam in the (θ,φ) = (0°, 0°) direction. Similarly, the corresponding results of (θ,φ) = (30°, 0°) are depicted in Figure 7d. According to Figure 9e,f, it is observed that two RHCP waves are generated, and the centers of two vortex waves point to the (0°, 0°) and (30°, 0°) directions, respectively. Therefore, simulation results demonstrate that the spin-controlled OAM wave with various topological modes can be achieved in arbitrary spatial directions by the proposed graphene metasurface. This proposed generator can convert a circularly polarized incident wave into a spin-controlled vortex wave that possesses SAM and OAM simultaneously. Compared with the linearly polarized OAM beam, the proposed spin-controlled beam can provide an extra degree of freedom for THz applications. The proposed generator has good performance and a low profile, facilitating the integration of devices, which is very promising for future THz communication applications. 

## 5. Conclusions

In summary, a new multifunctional graphene metasurface based on the principle of PB phase to control the SAM and OAM of the THz reflected waves has been proposed and numerically demonstrated. Composed of a three-layer graphene structure by rotating orientations of graphene elements, the proposed graphene element not only achieves 360° phase coverage, but also possesses the capability of polarization maintenance for the CP wave. In order to verify the results of the unit cell in HFSS, another modeling tool, CST, was also used to simulate the same unit cell; meanwhile, the differences of the results were also analyzed. Then, a detailed design strategy was also provided to further help readers obtain a deeper insight into graphene-based metasurfaces. To demonstrate the performance of our proposals, three kinds of graphene metasurfaces with various functions, including independent control of SAM, OAM or both of them, i.e., a THz spin-controlled OAM beam generator, were designed and numerically demonstrated. Compared with a linearly polarized OAM beam, it is noted that the spin-controlled beam produced by our designs provides an extra degree of freedom for THz wireless communication and improves the capability of the channel without increasing the bandwidth, which has great potential for future high-speed THz communication systems.
